# The effect of antimicrobial peptide-added adhesive resins on shear bond strength and the adhesive remnant index of orthodontic brackets

**DOI:** 10.1186/s12903-024-04462-9

**Published:** 2024-07-20

**Authors:** Kevser Kurt Demirsoy, Suleyman Kutalmış Buyuk, Melek Hilal Kaplan, Umut Kokbas, Feridun Abay, Ceyda Ozen, Alper Akkaya

**Affiliations:** 1 Department of Orthodontics, Faculty of Dentistry, Nevsehir Haci Bektas Veli University, Nevsehir, Türkiye; 2 Department of Orthodontics, Faculty of Dentistry, Ordu University, Ordu, Türkiye; 3 Department of Restorative Dentistry, Faculty of Dentistry, Nevsehir Haci Bektas Veli University, Nevsehir, Türkiye; 4 Department of Biochemistry, Faculty of Dentistry, Nevsehir Haci Bektas Veli University, Nevsehir, Türkiye; 5 Department of Biochemistry, Science Faculty, Ege University, Izmir, Türkiye

**Keywords:** Antimicrobial peptide, Nisin, Shear Bond Strength, Adhesive Remnant Index, Orthodontics

## Abstract

**Objectives:**

The aim of this study was to evaluate the effect of *in-vivo* produced Nisin which is an antimicrobial peptide (AMP) added to adhesive resin on shear bond strength (SBS) and the adhesive remnant index (ARI) of orthodontic brackets.

**Methods:**

Bacterial AMP was produced by fermentation and the ideal AMP/Bond concentration and antimicrobial efficacy of the mixture were tested. To evaluate the SBS and ARI scores of AMP-added adhesive resins, 80 maxillary premolar teeth extracted for orthodontic purposes were used and randomly assigned into 2 groups (*n* = 40). Group 1: Control Group (teeth bonded with standard adhesive resin); Group 2: Experimental Group (teeth bonded with AMP-added adhesive resin). Statistical analysis was performed using the SPSS package program and applying the Mann-Whitney U and Fisher’s exact tests. *P* < 0.05 was considered as statistically significant.

**Results:**

Nisin synthesized *in-vivo* from *Lactococcus lactis (L. lactis) (ATCC 7962)* bacteria was provided to form a homogenous solution at an ideal concentration To find the minimum AMP/Bond mixture ratio that showed maximum antimicrobial activity, AMP and Bond mixtures were tested at various concentration levels between 1/160 and 1/2 (AMP/Bond). As a result, the optimum ratio was determined as 1/40. The antimicrobial efficacy of Nisin-added adhesive resin was tested against *Streptococcus mutans (S. mutans) (ATCC 35,688)* and *Lactobacillus strains* (cariogenic microorganisms). AMP formed a 2.7 cm diameter zone alone, while 1/40 AMP-bond mixture formed a 1.2 cm diameter zone. SBS values of the teeth bonded with Nisin added adhesive (17.49 ± 5.31) were significantly higher than the control group (14.54 ± 4.96) (*P* = 0.004). According to the four point scale, Nisin added adhesive provided a higher ARI score in favour of the adhesive and tooth compared to the control group (ARI = 3, *n* = 20).

**Conclusions:**

Nisin produced from *L. lactis (ATCC 7962)* had greater antimicrobial effects after mixing with adhesive bond against cariogenic microorganisms *S. mutans (ATCC 35,688)* and *Lactobacillus strains*. Nisin added adhesive increased shear bond strength (SBS) of orthodontic brackets and ARI scores in favor of adhesive & teeth.

**Clinical relevance:**

Clinicians should take into account that using Nisin-added adhesive resin in orthodontic treatments can provide prophylaxis against tooth decay, especially in patients with poor oral hygiene.

## Introduction

Fixed appliance orthodontic treatments are long-term applications that require continuous patient motivation. During treatment, demineralisation of enamel adjacent to brackets can occur due to a patient’s eating habits and deficient oral hygiene [1]. White spot lesions (WSL) are demineralisation zones below the enamel surface that are characterised by a reduction in mineral content, causing a more opaque appearance compared to intact enamel [2]. Studies have reported the incidence of new WSL during orthodontic treatment to be 45.8% and the prevalence in patients with previous orthodontic treatment to be 68.4%.^3^ The reported prevalence rates of WSL during orthodontic treatment vary between 2 and 97% in different epidemiological studies [4, 5]. Orthodontic treatment has been identified as a potential threat to public health due to treatment requirements and complications caused by biofilm accumulation [6]. 

Current treatment approaches for WSL focus on remineralisation to protect natural tooth tissues and prevent or delay interventions [7, 8]. In addition to tooth decay, buccal WSL can cause aesthetic concerns [9]. Various methods have been applied for WSL treatment, including antimicrobial toothpastes, laser applications, mouthwash, fluorides, casein phosphopeptides-amorphous calcium phosphate, and antimicrobial modifications of orthodontic bio-substances [10, 11]. While fluorides are effective in preventing WSL formation, their influence on reducing existing WSL is limited [12, 13]. Other remineralisation resins based on calcium phosphate have been studied, but no clinically significant superiority has been found over fluoride [12–15]. 

*Streptococcus mutans* and *Lactobacillus* are known cariogenic pathogens that cause tooth decay [16]. Antibiotics have been shown to reduce tooth decay, but routine antibiotic use for carious tooth treatment is not recommended due to the risk of bacterial resistance and a lack of remineralising properties [17]. Antimicrobial peptides (AMPs) have been suggested as an alternative to conventional antibiotics in managing caries because they can target the pathogens while protecting healthy oral microflora [18]. AMPs have broad-spectrum properties against various bacteria, fungi, and viruses, and are naturally present in the oral cavity [19]. It is also promising that cationic antimicrobial peptides develop less antimicrobial resistance against oral pathogens than antibiotics [20]. Synthetic AMPs have the potential to provide secure, effective, and ecologically balanced support to oral and tooth health [18]. Another holistic perspective to advance dental implantology with the use of AMPs; the potential of antimicrobial peptide coatings on dental implants aims to reduce peri-implantitis and improve success rates through enhanced osteoblast growth and prevention of bacterial infection [21]. 

The type and content of adhesive resins used in orthodontic treatment can also affect tooth decay and surface WSL formation [22]. Nanoparticles have been added to adhesive resins to improve their properties. However, adding antimicrobial nanoparticles to adhesive resins may weaken the bond strength between the adhesive resin and tooth tissue. There is currently only a single study which reported on the use of Nisin, an antimicrobial peptide, in orthodontic bonding procedures [23]. Therefore, the present study aimed to evaluate the effect of in-vivo produced, Nisin-added adhesive resin on the shear bond strength (SBS) of orthodontic brackets and the adhesive remnant index (ARI) following debonding.

## Materials and methods

### Study design

The study was evaluated and approved by the Nevsehir Haci Bektas Veli University Clinical Studies and Publication Ethics Committee (Decision no: 2021.09.364) and received support from the Nevsehir Haci Bektas Veli University Scientific Research Projects Coordination Unit (Project no: HDP21SAG1). Nisin was synthetically produced under in-vivo conditions at the Ege University Biochemistry laboratory, and its antimicrobial efficacy was evaluated. The sample size required to assess the effect of Nisin-added adhesive resin on shear bond strength (SBS) and adhesive remnant index (ARI) scores of orthodontic brackets was determined using G*Power software. It was estimated that 72 teeth would provide the trial with a 0.6 effect size and 95% power (control group: 36 teeth, experimental group: 36 teeth). To increase the reliability of the study results, a total of 80 maxillary premolar teeth extracted for orthodontic purposes were gathered as the study material and divided into 40 teeth for each group.

### Antimicrobial peptide synthesis and evaluation of antimicrobial efficacy

#### Nisin production

Nisin was produced by *Lactobacillus lactis* strains obtained from generated culture collections. MRS Broth medium was used to produce nisin by fermentation process from *Lactobacillus lactis* culture as like literature [24]. Once the desired incubation period was completed, the bacterial culture was harvested through centrifugation and filtration to separate the bacterial cells from the culture medium [25]. 

#### Synthesis of AMP-added adhesive resin

The AMP-added adhesive resin was produced by synthesising amino acids that bound to the acid end in an environment in which the amino acid N-terminal was preserved. The minimum inhibitory concentration against *S. mutans (ATCC 35,688)* and *Lactobacillus cariogenic microorganism strains* was determined to detect the optimal adhesive-antimicrobial peptide concentration. The synthesis followed the Fmoc solid-phase peptide synthesis principle [26]. 

#### Extraction and purification of nisin

The nisin was extracted from the harvested culture, followed by purification of the compound from other cellular components. Precise extraction and purification methods included solvent extraction, filtration, chromatography, or other specialised methods. The concentration and purity of the extracted nisin were measured using analytical techniques involving enzyme-linked immunosorbent assay (ELISA).

#### Purification of AMP

The AMP produced from *L. lactis (ATCC 7962)* (Cas No. 1414-45-5) bacteria was purified to synthesise nisin. Disc diffusion test was performed to determine the effectiveness of the purified antimicrobial peptide against the microorganism. In this test, the zone diameter determines the sensitivity of the microorganism to the antimicrobial agent used. The zone diameter obtained because of an antibacterial test applied to a sample taken from the AMP synthesis environment was 16 mm. After ammonium sulfate precipitation, the zone diameter increased to 20 mm. With Sephadex G75 column loading (gel filtration chromatography), the zone diameter reached 25 mm. Finally, with DEAE Sepharose Sephadex G75 column loading (ion exchange chromatography), the zone diameter increased to 27 mm [27]. 

#### Optimum conditions for synthesized nisin antimicrobial peptide

**Fig. 1:**
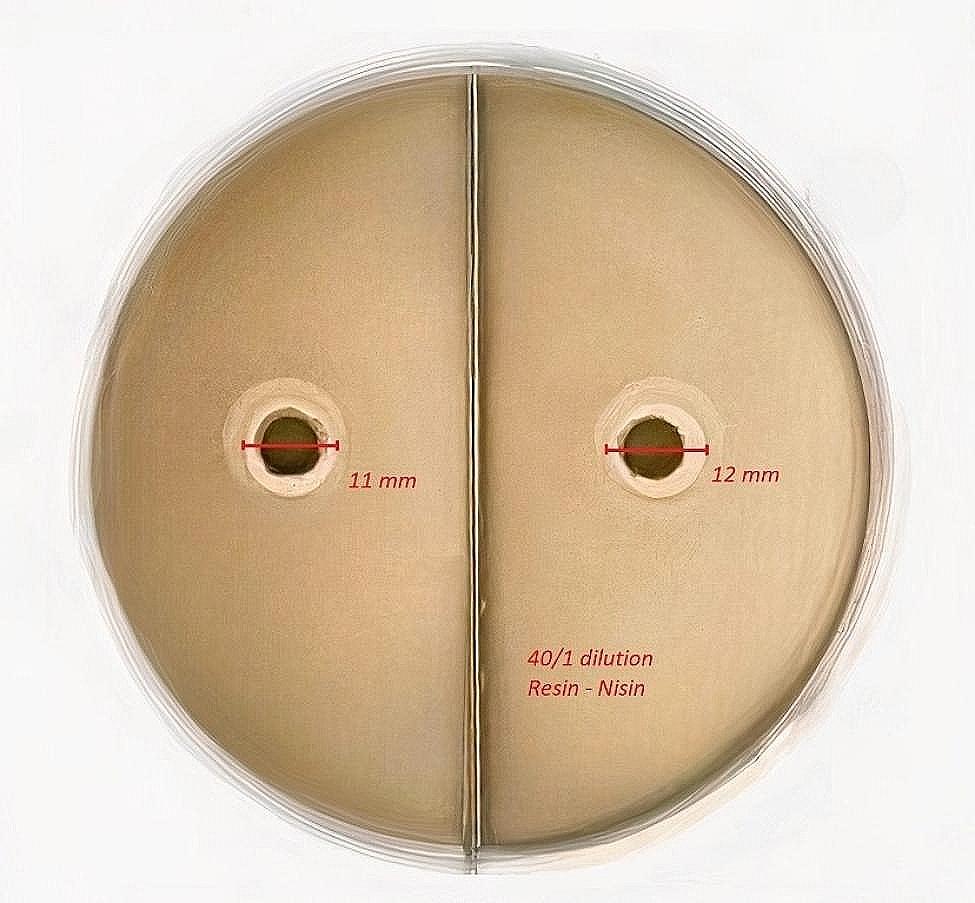
Zone diameters obtained with 1/40 Nisin AMP-bond (respectively) mixture rates

The conditions in which synthesized nisin antimicrobial peptide exhibited optimum efficacy were determined by variable manipulation, and it was found to be at pH 6.7. The antimicrobial efficacy was then tested at different concentrations (1–1/80) using scotch bond. The antimicrobial peptide alone formed a zone with a diameter of 2.7 cm, while the mixture of AMP-bond at 1/40 concentration formed a zone with a diameter of 1.2 cm (Fig. 1). The zone diameter values indicated minimal risk of tooth decay development.

#### Preservation of nisin in different solutions

A pilot study was conducted to determine the solution that exhibited the highest bond strength for nisin preservation. The buffer solution that provided the most suitable and highest SBS for AMP mixed with 1/40 bond and preserved in four different buffer solutions (sodium citrate pH = 3, sodium citrate pH = 4, sodium phosphate pH = 4, and medium sample) was found to be sodium citrate at pH = 3.

#### Antimicrobial effect test

Initially, culture media preparation involved sterilising nutrient agar. *Streptococcus mutans (S. mutans)* (ATCC 35,688) and Lactobacillus strains (cariogenic microorganisms) were inoculated onto the agar plates by streaking a standardised suspension of the microorganisms. The nisin was dissolved to achieve the desired concentration, and discs containing the nisin samples were placed on the surface of the agar plates. After incubation, the agar plates were examined for zones of inhibition, which appeared as clear areas around the nisin discs where the growth of microorganisms was inhibited. The diameters of the zones of inhibition were measured using a calliper [28]. 

Appropriate positive and negative controls were included in the assay. Positive controls consisted of discs impregnated with a known antimicrobial agent, while negative controls were discs or wells containing the solvent or diluent without nisin. The zone sizes of the nisin discs were compared with the positive and negative controls. The average diameter of the zones of inhibition for each concentration of nisin was measured, and the data analysed to determine the antimicrobial activity of nisin against *Streptococcus mutans (S. mutans*) (ATCC 35,688) and Lactobacillus microorganisms [29]. 

### Tooth preparation

A total of 140 upper premolar teeth extracted for orthodontic reasons were collected over a period of 4 months and preserved in distilled water containing 0.1% (weight/volume) thymol at room temperature. The inclusion criteria for the study teeth were an absence of any carious lesion, the absence of buccal enamel cracks or fractures, the absence of hypoplastic enamel tissue, the absence of restorations and developmental enamel disorders. Eighty teeth that met the inclusion criteria were accepted as the study material. The tooth surfaces were cleaned with pumice without fluoride (Ortho Teeth Cleaner, Shofu) and a rubber prophylaxis brush (Merssage Cup No.15, Shofu) for 15 s, rinsed with air-water spray, and dried before bonding the brackets to the tooth surface. The 80 teeth were randomly assigned to two equal groups (*n* = 40) identified as:

#### Group 1 (G1)

A Control Group (teeth bonded with standard adhesive resin resin without AMP).

#### Group 2 (G2)

An Experimental Group (teeth bonded with AMP-added adhesive resin).

The following materials were used: 0.022 inch slots mean bracket base area of 9.94 mm [2], Roth system, Mini Master premolar metal brackets (Mini Master Series, American Orthodontics, California, USA), Universal bond (3 M Espe, Scotchbond Universal Plus Bonding, Germany), 37% orthophosphoric acid (3 M™ ESPE™ Scotchbond™ Etchant Gel, 3007), Transbond XT bracket adhesive composite (3 M Unitek), and VALO™ Cordless light device. All materials were used according to the manufacturers instructions, and brackets were appropriately bonded to the teeth. The materials and contents used in the study are presented in Table 1.

**Table 1:** Contents, origins and serial numbers of materials used in the study

Materials	Lot number	Manufacturer	Country	Ingredients
Acid etch	220,686	BLUE HIGH VISCOSITY	USA	Contains Phosphoric Acid and Benzalkonium Chloride
Adhesive-1	10,450 A	3 M ESPE-Single Universal Bond	Germany	Ethyl Alcohol %25–35, Bisphenol A Diglycidyl Ether Dimethacrylate %10–20, Silane Treated Silica (Nanofiller) %10–20, 2-Hydroxyethyl Methacrylate %5–15, Glycerol 1,3-Dimethacrylate %5–10, Copolymer Of Acrylıc And Itaconic Acids %5–10, Diurethane Dimethacrylate %1–5, Water %<5
Adhesive-2	-------------	3 M ESPE-Single Universal Bond added to Nisin AMP (%13)	Türkiye	Ethyl Alcohol %25–35, Bisphenol A Diglycidyl Ether Dimethacrylate %10–20, Silane Treated Sılıca (Nanofiller) %10–20, 2-Hydroxyethyl Methacrylate %5–15, Glycerol 1,3-Dimethacrylate %5–10, Copolymer Of Acrylıc And Itaconic Acids %5–10, Diurethane Dimethacrylate %1–5, Water %<5,Nisin AMP %25
Composite	NE23926	Transbond XT,3 M Unitek	USA	—Bis-GMA, bis-FMA, acrylate, monomers, filler
Bracket	L-000373…	American Orthodontics Mini Master Metal Bracket	USA	Contains Nickel and /or Chromium
Curing Light	SNO05354	VALO ORTHO-CORDLESS	USA	Utilizable wavelength range: 385–515 Demetron L.E.D. Radiometer 1600nW/cm^2^

### Shear bond strength (SBS) test

**Fig. 2:**
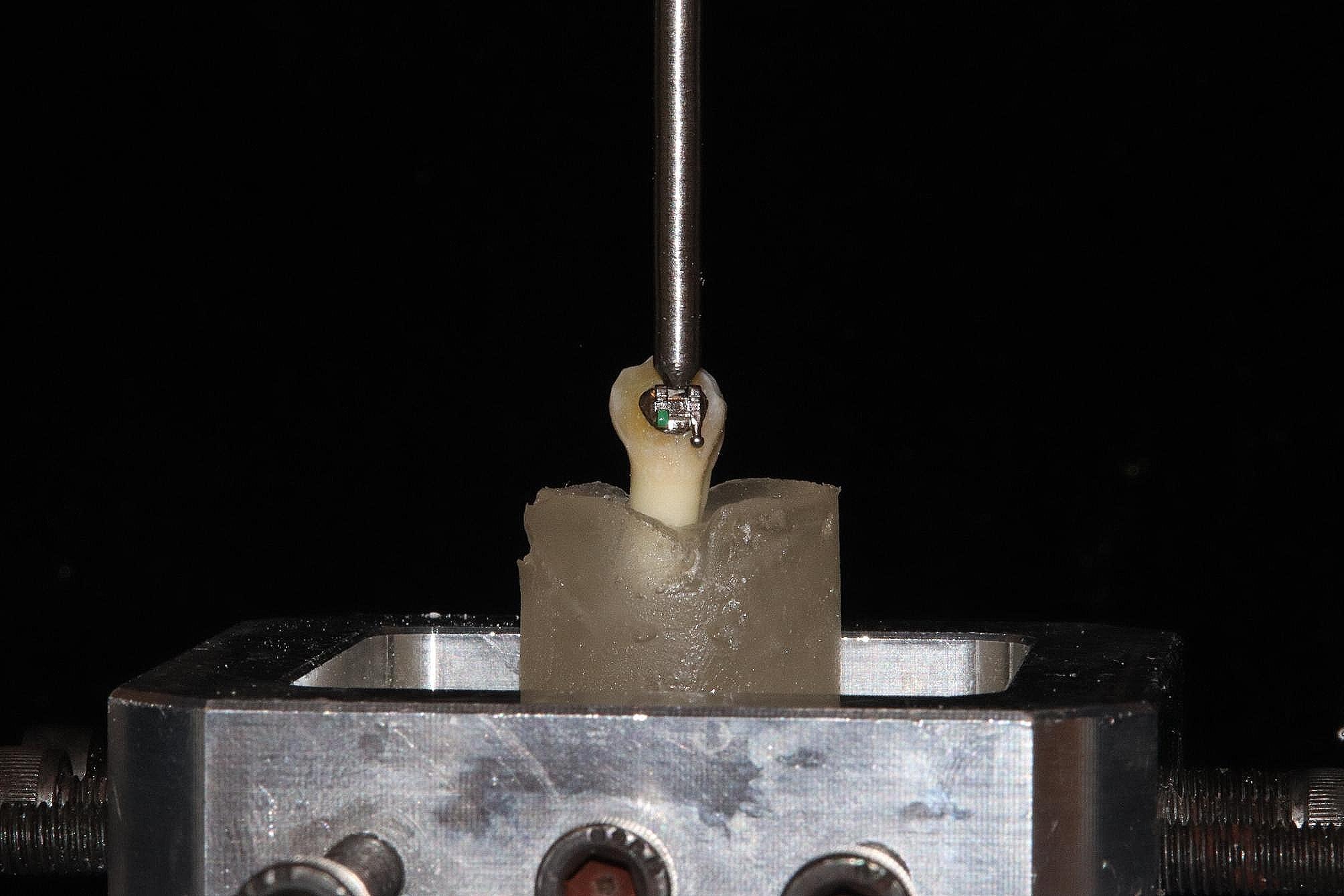
Shear bond strength of brackets using INSTRON device

After embedding the tooth samples in acrylic blocks, SBS measurements were performed using an INSTRON universal test machine (Z020; Zwick/Roell, Ulm, Germany). The SBS test was set up such that a 2.5 kg load was applied on the bracket base by a compression shear force in an occlusogingival direction (force applied perpendicular to the bracket base) (Fig. 2). The force required to separate the bracket from the tooth surface was recorded in Newtons at a crosshead velocity of 1.0 mm/min, and the SBS value was calculated using the following formula: [30, 31] Shear Bond Strength (SBS) Value = Force (N) / Bracket Base Area (mm [2]).

### Adhesive remnant index

**Fig. 3:**
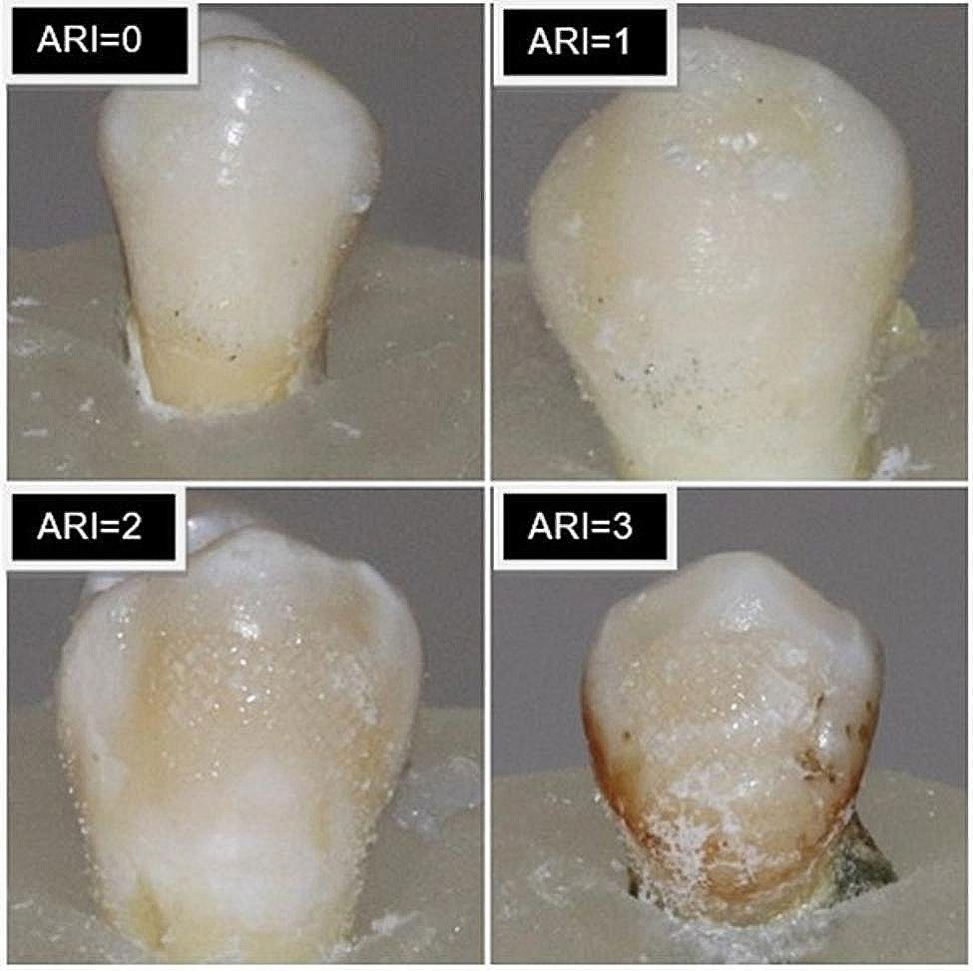
Adhesive remnant index according to ARI indices

After removing the brackets, photos of the bracket bases and buccal enamel tissues were taken using a stereomicroscope with 10x magnification (Stemi 305, ZEISS, Tokyo, Japan). The photos were recorded on a computer and numbered by an assistant who was not involved in the study to avoid analytical bias. The adhesive remnant index (ARI) was determined on the numbered photos by an experienced researcher using the method recommended by Bergland and Artun [32]. The ARI index ranged from 0 to 4, using the following interpretations:

- Index 0: No adhesive on the tooth surface,
- Index 1: Less than 50% adhesive on the tooth surface,
- Index 2: More than 50% adhesive on the tooth surface,
- Index 3: All adhesive on the enamel surface with bracket traces,
- Index 4: Fracture on the enamel surface (Fig. 3).

### Statistical analysis

Statistical analysis was performed using SPSS software (Windows version 20.0 for SPSS; SPSS Inc, Chicago, Illinois). After applying the normal distribution test, non-parametric tests (Mann-Whitney U test) were used for data that did not follow a normal distribution. Fisher’s exact test was used for the ARI index. A significance level of *P* < 0.05 was considered statistically significant.

## Results

The optimum concentration value, where the antimicrobial effect of nisin would minimize the binding, was found to be 1/40 nisin/resin (Fig. 1). To obtain this value, the nisin/resin mixture was tested using the serial dilution technique. Later, studies were carried out on the 1/40 nisin/resin complex.

The SBS values obtained from the experimental and control groups are presented in Table 2. The SBS values of the teeth bonded with Nisin-added adhesive resin (17.49 ± 5.31) were significantly higher than those of the control group (14.54 ± 4.96) (*P* = 0.004).

**Table 2:** Mean and standard deviations of SBS (MPa) values between groups

	Shear Bond Strength (MPa)				P*
	Mean (SD)	25p	Medium	75p	
Group 1 (Control)	14.54 (4.96)	11.92	13.57	17.21	**0.004**
Group 2 (Nisin AMP added adhesive)	17.49 (5.31)	14.12	16.68	20.93	

* Mann-Whitney U test results, SS; standard deviation, 25p; 25% percentile, 75p; 75% percentile

The distribution of ARI index between the groups is presented in Table 3. In half of the teeth bracketed using Nisin-added adhesive resin, all the adhesive remained on the enamel surface after debonding, and bracket traces were found (*N* = 20). There were no teeth with an ARI index of 0. In the control group, there was no adhesive remnant on 5 teeth surfaces (ARI = 0), while the ARI index was 3 in 16 teeth. The Nisin-added adhesive resin group had a total of 38 teeth with ARI indices of 2 and 3, whereas the control group had 25 teeth. No enamel surface fractures were found in either group (ARI = 4).

**Table 3:** Distribution of ARI indices among groups

	ARI Indices				*N*	P*
	*0*	*1*	*2*	*3*		
Group 1 (Control Group)	5	10	9	16	40	**0.002**
Group 2 (Bond with Nisin AMP containing)	0	2	18	20	40	

* Fischer’s Exact test results

## Discussion

Preventing disease related to dental treatment is the most mandatory aim from a conservative and cost perspective. During orthodontic treatment, it is important to focus on preventive methods against white spot lesions (WSL) and tooth decay, modify risk factors, and develop strategies for lesion remineralisation [1]. Recent approaches include the use of topical fluoride, amorphous calcium phosphate, a combination of agents, microcrystal hydroxyapatite (HAP) particles, and antimicrobial peptide (AMP) applications. (33–34)

Antimicrobial peptides are components of host defence proteins that act against microbial invasion through various mechanisms [34, 35]. Many studies have examined the mechanism of peptide secretion and the prevention of bacterial activity [35–38]. In the present study, Nisin, a bacteriocin of the lantibiotic class produced by *L. lactis (ATCC 7962)*, was combined with adhesive bond to examine its efficacy against *S. mutans* (ATCC 35,688) and Lactobacillus strains. Nisin binds to lipid II, which is involved in cell wall biosynthesis, thereby preventing bacterial membrane formation [37]. It has a wide antibacterial effect against Gram-positive bacteria, including Staphylococcus, Streptococcus, and Clostridium [38, 39]. The present study evaluated the efficacy of Nisin against oral cariogenic bacteria and determined the minimum effective concentration (MEC) of the adhesive-peptide mixture.

Natural AMPs are produced by salivary glands and epithelia in the oral cavity and play a role in the immune system [35]. However, natural AMPs have limitations in clinical applications, due to the high cost of synthesis and the reduction of long-term antimicrobial efficacy in saliva. Synthetic AMPs have been studied to overcome these limitations [13, 14]. In the present study, Nisin was biosynthetically produced from *L. lactis (ATCC 7962)* bacteria and preserved in four different buffer solutions (Sodium citrate pH = 3, Sodium citrate pH = 4, Sodium phosphate pH = 4, and medium sample). The optimal buffer solution was determined to be Sodium citrate pH = 3.

The shear bond strength (SBS) values of brackets bonded with Nisin-added adhesive resin were significantly higher than the control group, contradicting the expectation that SBS decreases with impaired adhesive resin purity. This indicates that the Nisin-added adhesive resin had both antimicrobial effects and strong bonding properties. This combination of increased SBS and antimicrobial effects may be advantageous when treatment duration is considered and in the prevention of WSL, a common complication of long-term orthodontic treatment. A comparison of the results with previous studies was not possible as no literature was found which examined the effect of Nisin-containing bond resins on SBS indices of orthodontic brackets.

Reynolds [40] suggested that an SBS value of 5.9–7.8 MPa for bracket bonding in orthodontic treatments is sufficient. In the present study, both the control group (14.54 ± 4.96) and the experimental group (17.49 ± 5.31) had higher SBS values than those previously reported, indicating ideal bonding.

Partial acid etching was performed slightly above the bracket base in this study. It has been suggested that total and partial acid etching principles do not differ significantly in assessing WSL formation, but less bracket failure is expected within 6 months following partial acid etching [41]. Pure Nisin formed a 2.7 cm diameter zone during antimicrobial tests, while the Nisin-adhesive mixture formed a 1.2 cm (12 mm) diameter zone at a 1/40 mixture rate. This zone diameter is believed to provide a safe antimicrobial and anti-cariogenic effect zone on the tooth surface when applying the adhesive under partial acid etching. The zone diameter values are expected to reduce the risk of WSL formation in the application zone. Total acid etching, however, may provide efficacy in the gingival regions due to the creation of wider antimicrobial distribution.

The role of bracket type in increasing bonding strength to composite resins was evaluated by Lai et al. [42], who found that bracket type was a more important factor than adhesive type. An additional study comparing SBS indices according to bracket type showed that ceramic brackets had significantly higher SBS indices than metal brackets [43]. Stainless steel metal brackets were used due to advantages related to ease of application, cost, widespread use, and generalisability of results to clinical conditions, despite their aesthetic limitations [30]. 

A higher adhesive remnant index (ARI), indicating weak bonding between the bracket and composite, is preferable because it reduces the likelihood of enamel cracks during debonding [44]. In the present study, more teeth had ARI = 3 scores in the group bonded with Nisin-added adhesive resin than in the control group, indicating higher enamel-adhesive SBS. No teeth had an ARI = 0 score. The Nisin-AMP content is believed to enhance the establishment of an organic bond with the tooth. However, high ARI scores increase the orthodontist’s workload and exposes the enamel surface to abrasion during the debonding process.

In vitro studies aim to mimic clinical conditions as closely as possible and simulate the oral cavity to provide objective results applicable to clinical conditions. However, it is important to consider variables identified as tooth collection time, preservation conditions, application method and duration of treatment resins, adhesive type, and bracket base type, which could influence the results [45]. In vitro studies can guide clinical applications and provide valuable insights before clinical use. The present research serves as a pilot study before active clinical applications, with the potential to develop homogenous peptide-bond mixture adhesive resins that can be safely used in clinical settings after cytotoxicity tests. The use of nisin-added adhesive resin offers advantages associated with antimicrobial efficacy, the prevention of WSL formation, and increased bond strength.

The oral cavity pH varies depending on eating habits. Nisin, being resistant to low pH, is expected to maintain its antimicrobial efficacy on tooth surfaces affected by different dietary habits. Due to its preservative properties, Nisin is frequently used in the food industry [46]. It is anticipated its oral efficacy would be retained during orthodontic treatment. Further in vitro studies may be designed to test the long-term antimicrobial efficacy of Nisin-containing bond resins under conditions that mimic the oral cavity. The SBS scores of brackets bonded with Nisin-added adhesive bond on tooth surfaces with restorations such as composite and porcelain may also be evaluated. Additionally, long-term clinical studies are needed to examine the persistence of biosynthetically produced Nisin-AMP and its antimicrobial efficacy compared to other synthetic counterparts in the oral environment.

After conducting a series of cytotoxicity tests, the use of Nisin-containing bond resins in clinical applications may help prevent the formation of white spot lesions and early tooth decay as significant side effects associated with orthodontic treatment. The routine use in orthodontic practice can enhance the antimicrobial efficacy against *S. mutans* (ATCC 35,688) and Lactobacillus strains. Although the high ARI scores increase the workload of the orthodontist and expose the enamel surface to abrasion during debonding, the advantages of Nisin-added adhesive resin, including increased bond strength and efficacy at low pH, can prevent bracket failure and unwanted extension of treatment time, independent of a patient’s hygiene and dietary habits.

Apart from Nisin, there are other AMPs that may be biosynthetically reproducible and naturally present in the oral cavity. The treatment effect of AMPs on early lesions causing tooth decay can be utilised due to their remineralising effect. Further in vitro studies may be designed to test the long-term antimicrobial efficacy of AMP-containing bond resins in environments that mimic the oral cavity. Additionally, further clinical studies may reveal the efficacy of Nisin in other orthodontic treatments involving removable orthodontic appliances, orthodontic wires, and mini screws.

## Conclusions

After being produced from *L. lactis (ATCC 7962)* bacteria, Nisin, when mixed with adhesive bond, maintained its antimicrobial efficacy. Nisin-added adhesive bond resin increased the shear bond strength of orthodontic brackets. Nisin-added adhesive resin showed higher ARI scores in favour of the adhesive and tooth interface compared to the control group. Once the clinical feasibility is investigated at the end of cytotoxicity studies, long-term clinical studies may enable the routine use of Nisin-added adhesive resins in orthodontic practice.

## Data Availability

The datasets used and/or analyzed during the current study are available from the corresponding author on reasonable request.
